# Robust and tunable itinerant ferromagnetism at the silicon surface of the antiferromagnet GdRh_2_Si_2_

**DOI:** 10.1038/srep24254

**Published:** 2016-04-07

**Authors:** M. Güttler, A. Generalov, M. M. Otrokov, K. Kummer, K. Kliemt, A. Fedorov, A. Chikina, S. Danzenbächer, S. Schulz, E. V. Chulkov, Yu. M. Koroteev, N. Caroca-Canales, M. Shi, M. Radovic, C. Geibel, C. Laubschat, P. Dudin, T. K. Kim, M. Hoesch, C. Krellner, D. V. Vyalikh

**Affiliations:** 1 Institute of Solid State Physics, Dresden University of Technology, Zellescher Weg 16, D-01062 Dresden, Germany; 2 CSNSM, University Paris-Sud and CNRS/IN2P3, Bâtiments 104 et 108, 91405 Orsay, France; 3 MAX IV Laboratory, Lund University, Box 118, 22100 Lund, Sweden; 4 Donostia International Physics Center (DIPC), Departamento de Fisica de Materiales and CFM-MPC UPV/EHU, 20080 San Sebastian, Spain; 5 Tomsk State University, Lenina Av., 36, 634050 Tomsk, Russia; 6 European Synchrotron Radiation Facility, 71 Avenue des Martyrs, Grenoble, France; 7 Kristall- und Materiallabor, Physikalisches Institut, Goethe-Universität Frankfurt, Max-von-Laue Straße 1, 60438 Frankfurt am Main, Germany; 8 IFW Dresden, P.O. Box 270116, D-01171 Dresden, Germany; 9 Max Planck Institute for Chemical Physics of Solids, Nöthnitzer Strasse 40, D-01187 Dresden, Germany; 10 Swiss Light Source, Paul Scherrer Institute, CH-5232 Villigen-PSI, Switzerland; 11 SwissFEL, Paul Scherrer Institut, CH-5232 Villigen PSI, Switzerland; 12 Saint Petersburg State University, Saint Petersburg 198504, Russia; 13 Institute of Strength Physics and Materials Science, RAS, 634021 Tomsk, Russia; 14 Diamond Light Source, Didcot OX11 0DE, UK; 15 IKERBASQUE, Basque Foundation for Science, 48011 Bilbao, Spain

## Abstract

Spin-polarized two-dimensional electron states (2DESs) at surfaces and interfaces of magnetically active materials attract immense interest because of the idea of exploiting fermion spins rather than charge in next generation electronics. Applying angle-resolved photoelectron spectroscopy, we show that the silicon surface of GdRh_2_Si_2_ bears two distinct 2DESs, one being a Shockley surface state, and the other a Dirac surface resonance. Both are subject to strong exchange interaction with the ordered 4*f*-moments lying underneath the Si-Rh-Si trilayer. The spin degeneracy of the Shockley state breaks down below ~90 K, and the splitting of the resulting subbands saturates upon cooling at values as high as ~185 meV. The spin splitting of the Dirac state becomes clearly visible around ~60 K, reaching a maximum of ~70 meV. An abrupt increase of surface magnetization at around the same temperature suggests that the Dirac state contributes significantly to the magnetic properties at the Si surface. We also show the possibility to tune the properties of 2DESs by depositing alkali metal atoms. The unique temperature-dependent ferromagnetic properties of the Si-terminated surface in GdRh_2_Si_2_ could be exploited when combined with functional adlayers deposited on top for which novel phenomena related to magnetism can be anticipated.

Silicon-terminated surfaces of crystalline solids are intrinsically part of conventional electronics, but their exploitation in novel materials combining two-dimensional electron states (2DESs) and magnetism, which play an important role in the development of next-generation electronics, still remains elusive^1^. The appearance of 2DESs at surfaces or interfaces and their interplay with magnetic degrees of freedom may open an avenue for new physics in silicon-based technologies for future devices^1^. In our world, a natural source of very strong magnetism is elemental Gd, which contains a half-filled 4*f* shell^2^. Its ground state has a large pure spin moment J = S = 7/2 with vanishing orbital moment L = 0. Thus, in crystalline solids Gd will be insensitive to crystal-electric-field effects, which may strongly affect the magnetic properties of materials^3^. In the present work, we consider the layered antiferromagnet GdRh_2_Si_2_, which crystallizes in the tetragonal body-centered ThCr_2_Si_2_ structure^4^,^5^. Below the Néel temperature T_N _~ 107 K, the Gd 4*f* moments become ferromagnetically ordered within the *ab*-plane, while they stack in antiferromagnetic (AFM) order along the *c*-axis^4^,^6^. The Gd planes are well separated from each other by Si-Rh-Si trilayers. Similarly to other RERh_2_Si_2_ (RE = Yb, Eu) crystals^7^,^8^, the chemical bonds within the trilayer are much stronger than those between the Gd and Si plane, therefore the surface of a cleaved GdRh_2_Si_2_ crystal can be terminated either by Si or Gd atoms. Silicon termination is particularly interesting, since in this case the first magnetically active layer of Gd is hidden and protected by the Si-Rh-Si buffer at the surface. This leaves us with the question, what happens to the two-dimensional electrons confined at the silicon surface, when the 4*f* moments of the underlying Gd layer are ferromagnetically ordered, as it is schematically illustrated in Fig. 1.

As we will show below, the silicon surface of GdRh_2_Si_2_ reveals a remarkable property. Studying this system with angle-resolved photoelectron spectroscopy (ARPES)^9^, we find two distinct 2DESs arising from Shockley and Dirac fermions, which reveal surface- and surface-resonant behavior, respectively. The surface electronic states theoretically predicted by I. Tamm^10^ and later on by W. Shockley^11^ were observed on the (111) surface of noble metals in ARPES measurements^12^,^13^. These states, usually called Shockley states, lie exclusively in the bulk projected band gaps and in real space they are localized in a few surface atomic layers. Therefore they carry intrinsic quasi-2D electronic properties. In the recent past, Shockley states found on many metal and semiconductor surfaces attracted considerable attention due to their rich and exotic properties^9^,^14^. Surface resonance states also have a quasi-2D nature, but in difference to the Shockley states they can penetrate into the material thus overlapping with the bulk band states. The wave function of the surface resonance in the bulk becomes essentially modified at the surface and is characterized by an enhanced probability density in the near surface region. Surface resonances could have notable **k**_z_ dispersion^8^ and can be considered as a kind of bridge connecting the properties at the surface and in the bulk of the material^9^,^15^.

Here, we will focus on the linear-dispersive Dirac cone band, which appears at the 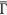-point, and on a parabolic Shockley surface state, which can be seen within a large gap in the projected bulk bands around the 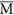-point i.e. at the corner of the surface Brillouin zone. Our ARPES study shows that when the Gd 4*f* moments become ordered, both spectral structures are spin-split and form well-defined subbands. We have investigated the momentum-resolved temperature evolution of the spin splitting for both spectral features by ARPES and support our discussion of their origin and nature with theoretical calculations and X-ray magnetic linear dichroism (XMLD) measurements. We conclude that the two distinct 2DESs being an intrinsic signature of the Si-terminated surface of GdRh_2_Si_2_ exhibit itinerant magnetism at the surface. Their spin splitting arises from the strong exchange interaction with the ordered Gd 4*f* moments lying below the Si-Rh-Si buffer. The temperature dependence of the spin splitting can be straightforwardly explained within the framework of conventional mean-field theory of the Heisenberg model^16^. Our results suggest that the ferromagnetic Si-terminated surface of GdRh_2_Si_2_ can serve as a model substrate to induce non-trivial electronic and magnetic properties into nanostructures deposited on top which could eventually become interesting for technological applications. We present a simple example of tuning the electronic properties of the Si-terminated magnetic surface by depositing alkali metal atoms. As a result, strong energy shifts of the surface states due to the electron doping effect of the topmost layers as well as an interplay of spin-split bands of the Shockley state and surface resonances were observed, implying controllable modification of magnetism at the surface and sub-surface regions of the material. In the recent past, we have demonstrated that the Dirac fermion states can couple with ultra-heavy quasiparticles in crystalline 4*f*-based systems^8^ by coupling to the electronic degree of freedom of 4*f* electrons. In this work, we show how the Dirac fermions can couple to the magnetic degree of freedom of 4*f* electrons.

## Results

### The coexistence of spin-polarized 2DESs at the silicon surface from theory and ARPES

**Figure 2:**
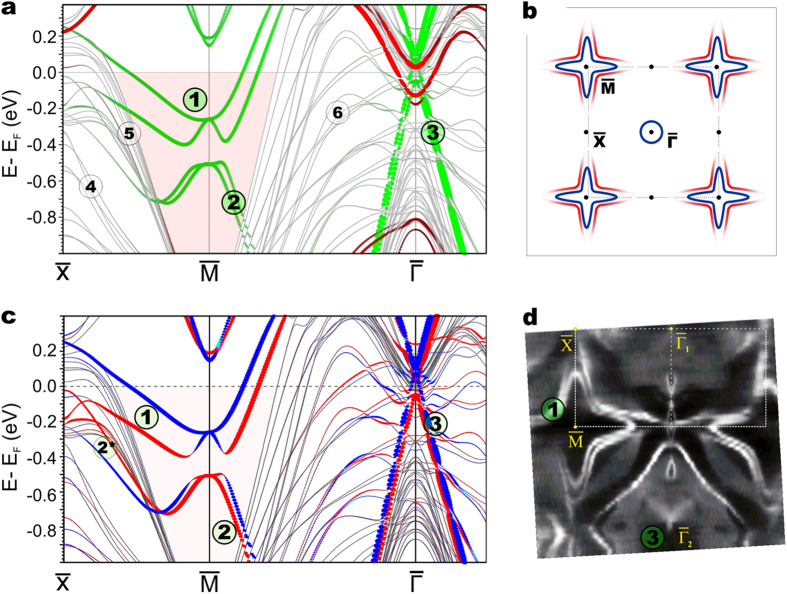
Surface-related electronic structure of AFM ordered GdRh_2_Si_2_. (**a**) Calculated electronic band structure for a slab of AFM ordered GdRh_2_Si_2_. The surface electron bands are displayed in red for the Gd- and green for the Si-terminated surface. Spin-split electron- and hole-like bands of the Shockley surface state at the 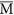
- projected band gap are marked by (1) and (2), respectively while the Dirac cone bands seen at the 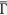-point are labelled as (3). Bulk-like projected bands are shown in gray and labelled as (4), (5) and (6). (**b**) A rather schematic view of the Fermi surface for the discussed 2DESs at the center of the Brillouin zone and at the 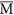
- point. Note that the lower of the two spin-split bands (1) of the Shockley state seen in (**a**) does not reach the Fermi energy along the 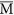-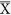 direction. (**c**) Calculated spin-resolved electronic band structure for the Si-terminated GdRh_2_Si_2_ surface (the contribution of the topmost Si-Rh-Si-Gd block to the spin vector components is shown). Majority/minority bands are shown in red/blue. The spin-polarized 2DESs are labelled in accordance with Fig. 2a. (**d**) ARPES-derived Fermi surface for AFM ordered and Si-terminated GdRh_2_Si_2_ taken at a temperature of 1 K using 45 eV photons.

In Fig. 2a we show the surface electronic band structure obtained from our *ab initio* calculations for AFM ordered GdRh_2_Si_2_. To separate the surface-related electronic structure from bulk electron bands, we used a thick slab which was terminated by Gd on the one and Si on the other side. This allows us to trace simultaneously bulk-like bands, band gaps and surface-related states for both terminations. The 2D electron states that are related to the Si-terminated surface are shown in green, while the respective bands for the Gd-terminated surface are highlighted in red. In Fig. 2b we rather schematically illustrate the Fermi surface of the two-dimensional states, which will be further discussed. The spin texture of the slab-derived electronic states for the Si-terminated surface of AFM ordered GdRh_2_Si_2_ is shown in Fig. 2c. For comparison, we also show the ARPES-derived Fermi surface for Si-terminated GdRh_2_Si_2_ taken at a temperature of 1 K in Fig. 2d. The presented non-symmetrized ARPES data allow to trace the evolution of photoemission intensity due to matrix element effects when going from the first to the second Brillouin zone (BZ).

When the crystal is terminated by a silicon layer, pairs of strongly dispersive bands appear in the large projected band gap around the 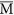-point labelled as (1) and (2) (Fig. 2a,c). Looking closely on the behavior of these bands within the 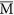-point gap we can conclude that both the majority and minority states (Fig. 2c) are degenerate at the 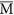-point and reveal a gap due to spin-orbit interaction. The observed spectral structure inside the 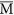-gap arises from Shockley surface electron states confined within the topmost few atomic layers of the Si-terminated crystal (see next section). The essential point is that this surface state is missing at the Gd-terminated surface. In the experimentally derived Fermi surface (Fig. 2d), the respective feature can be seen as a diamond-like structure wrapping the 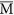-point. Exploring further the spectral structure within the 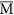-gap and along the 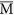-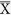 direction one can do a few more interesting observations. The electron-like spin-splitted lower subband of feature (1) does not reach the Fermi level and leaves the 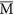-gap around 0.2 eV of binding energy (BE) (Fig. 2a,c). In comparison with the experimental data in Fig. 2d one may assume that this spectral feature may have a surface resonant behavior when it moves out of the 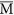-gap and approaches the 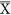-point. In the same region near the 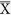-point another surface resonance band appears marked as (2*) which keeps its spin polarization up to the 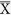-point. This band has been also detected in ARPES measurements which will be discussed below.

At the silicon surface, another remarkable feature labelled as (3) can be seen at the 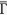-point, which apparently reveals a split pair of linear bands. The linear-dispersive conical shape corresponds to Dirac fermions and is imposed by the two-dimensional square symmetry of the layered crystal structure^8^,^17^,^18^. A similar Dirac cone band has been seen in homologous systems like EuRh_2_Si_2_^8^, YbRh_2_Si_2_^7^,^19^,^20^,^21^ and YbCo_2_Si_2_^22^ where its properties and interplay with the 4*f* states have been characterized.

Thus, the results of our slab band structure calculations clearly point out the coexistence of two 2DESs at the silicon surface of GdRh_2_Si_2_. Moreover, our calculations suggest that in AFM ordered GdRh_2_Si_2_ spin degeneracy is lifted for both Shockley and Dirac cone states (Fig. 2c). In order to verify this prediction and to study how this phenomenon is related to the magnetic ordering in the system, we explored experimentally these electron states, their band splitting and their temperature dependence.

**Figure 3:**
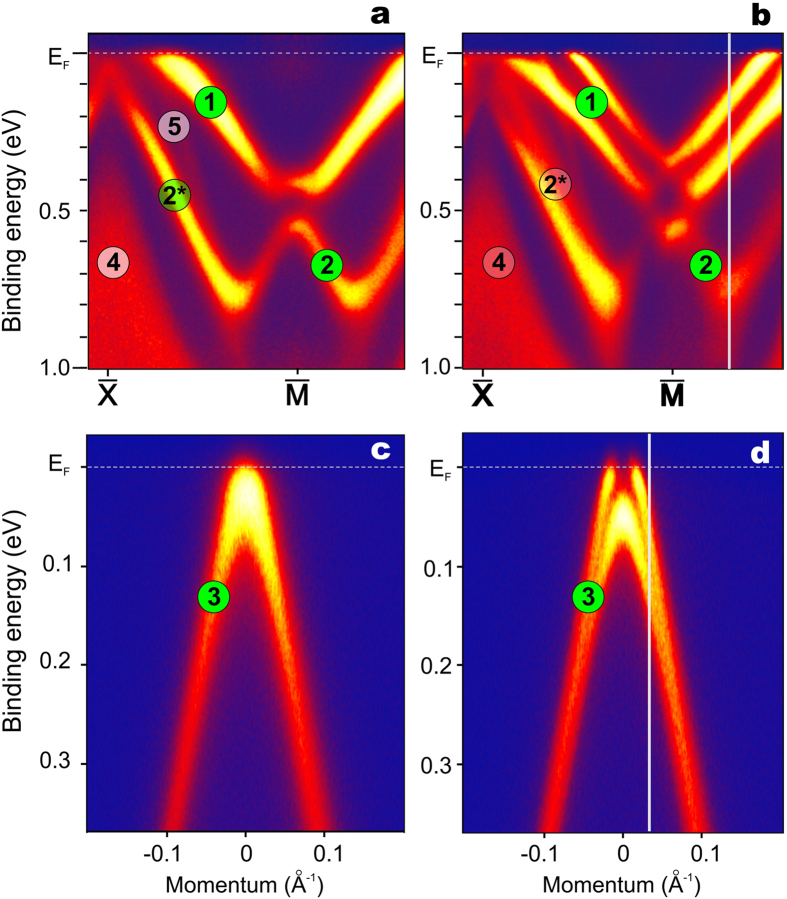
Spin splitting of the Shockley state and Dirac cone. ARPES data taken from a Si-terminated GdRh_2_Si_2_ sample using 55 eV photons. The band maps were obtained near the 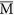-point at 117 K (**a**) and 19 K (**b**) and near the 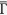-point at 72 K (**c**) and 19 K (**d**). The measurements were performed along the 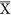-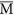 and 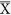-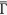 directions, respectively. The white vertical lines indicate the energy-distribution curves, which were further used for the analysis of the spin splitting in the T-dependent studies. The surface- and bulk-related spectral features are labelled in accordance to the theoretically derived bands seen in Fig. 2.

Surface-sensitive ARPES is ideally suited to explore the momentum-dependent electronic structure of a 2DES trapped at a crystal surface^9^. In Fig. 3 we present the ARPES-derived spectral structure of a Si-terminated GdRh_2_Si_2_ surface as a function of temperature around the 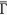
- and the 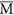-points of the surface Brillouin zone. The bulk Néel temperature T_N_ ~ 107 K marks the onset of in-plane ferromagnetic alignment of the Gd 4*f* moments, which stack antiferromagnetically along the *c*-axis^4^. We thus cleaved the crystal at 120 K well above T_N_. Si termination can be easily identified by the presence of the intense Shockley surface state at the 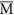-point, which is absent for Gd termination. The high-temperature band map in the paramagnetic state is shown in Fig. 3a. Comparing the ARPES data to the results of our calculation, we can see that the Shockley state is unsplit. This changes dramatically when gradually cooling down the sample. To monitor the emergence of the spin splitting, we choose an energy distribution curve (EDC) at **k**_||_ ~ 1/3 of the 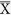-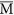 distance near the 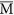-point as indicated by the vertical line in Fig. 3b. With bare eyes, the appearance of the spin splitting becomes visible near ~90 K notably below the Néel temperature. Upon further cooling, the splitting becomes well resolved and its value rapidly increases, reaching ~160 meV. We have similarly followed the spectral structure of the Dirac cone at the 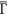-point as a function of temperature, using the EDC at **k**_||_ marked by the vertical line in Fig. 3d. In this case, the band splitting becomes apparent only at temperatures below ~70 K. Figure 3c shows the spectral pattern at 72 K without evident spin splitting, whereas in Fig. 3d the splitting is nicely seen at 19 K with a maximum value of ~70 meV. A detailed analysis of the measured temperature dependence of the band splitting will be given below.

### Dirac cone and Shockley state: Temperature dependence of their spin splitting

**Figure 4:**
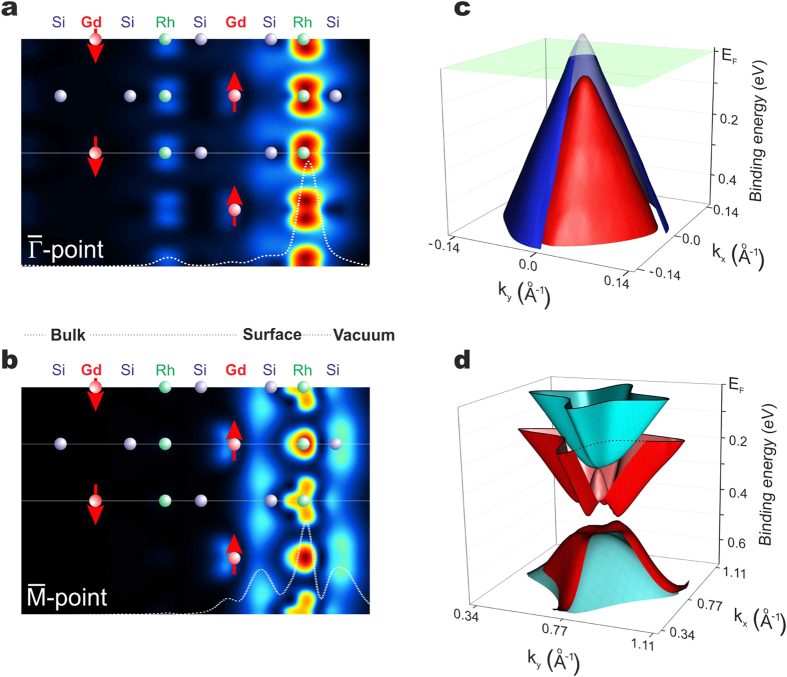
Theoretical insight into the spin-split Dirac cone and Shockley state. Electron density distribution (projected on the *ac*-plane) of the Dirac cone near the 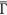-point (**a**) and the Shockley state at the 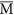-point (**b**). Dotted lines show the respective electron density distributions integrated over the *ab*-plane. Three-dimensional representation of the theoretically derived spin-split Dirac cone (**c**) and the Shockley state (**d**) calculated for the AFM phase of GdRh_2_Si_2_.

A deeper understanding of the properties of the discussed 2DESs can be obtained from the analysis of the orbital composition and visualization of the spatial extension of the calculated eigenstates. Representative electron densities of the Dirac cone near the 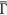-point and the Shockley state at the 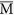-point are shown in Fig. 4a,b, respectively. An analysis of the orbital character of the 2DESs reveals that the Shockley state is built up mainly by Si 3*s* and 3*p* (53%), Rh 4*d* (30%) and Gd *5d* (14%) states. Our calculations suggest that the Shockley state is located exclusively within the first four atomic layers Si-Rh-Si-Gd and only 3% of this state penetrates in the vacuum. The Dirac cone is essentially built by Rh 4*d t*_*2g*_ (74%) orbitals with an admixture of the Si 3*p*_*x,y*_ states (23%) and a tiny Gd 5*d* (3%) contribution. Its conical structure is a consequence of the in-plane square symmetry of the layered material^8^,^17^,^18^. The fourfold warping of the Dirac cone can be well recognized in its three-dimensional representation derived from our calculations (Fig. 4c). The theoretically derived Shockley state that is shown in Fig. 4d, is in excellent agreement with our ARPES results: the spin splitting of the electron- and hole-like bands as well as the appearance of the spin-orbit gap at the 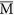-point are nicely reproduced.

Despite the 2D character of the Dirac cone, the electron density distributions in Fig. 4a,b show that its wave function penetrates deeper into the bulk than the Shockley state which is more strongly confined to the topmost four layers. In contrast to the Shockley state, the Dirac cone has the character of a surface resonance^9^. Its wave function is resonantly enhanced at the surface but keeps a finite contribution in the bulk having therefore notable **k**_z_ dispersion^8^. We have recently explored this property in details for EuRh_2_Si_2_^8^. The Shockley state decays exponentially into the crystal and is well localized at the surface region. We have verified that with increasing slab thickness, the orbitals from atoms below the first Gd layer and further in the bulk, mainly from Rh 4*d*, contribute to the Dirac cone, confirming its resonant nature. Moreover, the band structure in Fig. 2a,c shows that the Dirac cone essentially overlaps with the bulk bands, which appear as a bunch of gray lines in the slab calculation (Fig. 2a), where small hybridization gaps open up at the band crossings. However, both 2DESs show a spin splitting as a consequence of the exchange coupling to the Gd 4*f* moments. One may thus anticipate that the fundamental differences between the Dirac cone and the Shockley state substantially affect the strength of the exchange interaction with the ordered Gd 4*f* moments near the silicon surface and the temperature evolution of the splitting.

**Figure 5:**
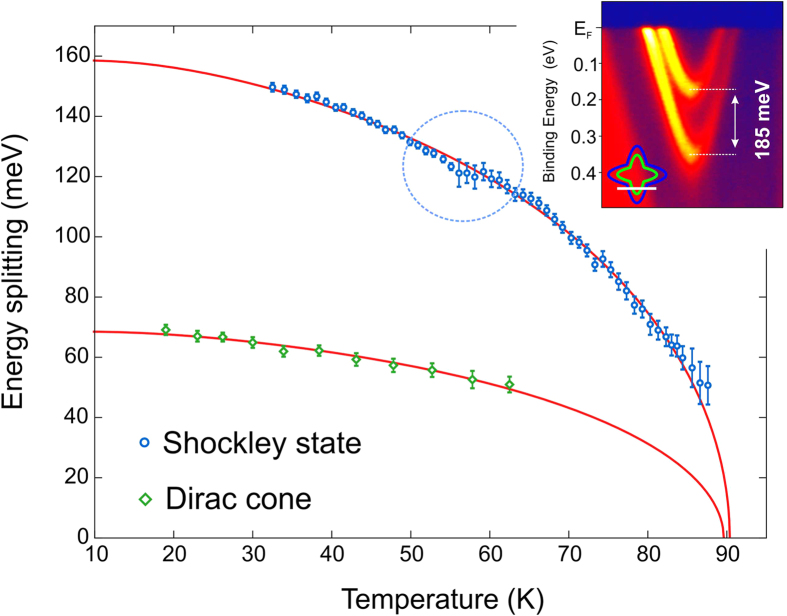
Temperature dependence of the spin splitting. ARPES-derived temperature evolution of the spin splitting for the Shockley state and Dirac cone. The solid lines represent the results of the fit analysis for both sets of data obtained by means of the Weiss molecular-field approximation to the Heisenberg model^16^. The inset shows the largest obtained spin splitting for the Shockley state reaching a value of 185 meV and schematically illustrates the direction of measurements.

In Fig. 5, we present the evolution of the spin splitting for both 2DESs as a function of temperature. The splitting values have been deduced from fits of the energy distribution curves, which have been chosen slightly away from the Fermi level crossing and in the case of the 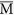-point well away from the spin-orbit gap (vertical lines in Fig. 3b,d). A few exemplary EDCs and details about the fitting procedure can be found in Supplementary Fig. S1. For the Shockley state, the splitting sets in at a temperature of ~90 K and rapidly approaches to a value of ~160 meV. At higher temperatures, the splitting seems to be much smaller than life-time and instrumental broadening and therefore cannot be resolved. Note that the monotonous increase of the splitting with decreasing temperature is interrupted by a “kink” around 60 K highlighted by a dotted circle, which will be discussed further. We found that the splitting of the Shockley state is actually anisotropic with values ranging from ~160 meV up to the largest observed value of 185 meV (inset in Fig. 5) in a direction parallel to 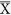-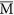 slightly away from the 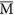-point. The experimentally established spin splitting of the Shockley state is in agreement with the results of our theoretical studies. Interestingly, the averaged spin splitting of this state is larger than that observed for EuRh_2_Si_2_ (~150 meV^23^), but not as much as one might anticipate.

To model the temperature evolution of the Shockley state splitting, we fit the data with a magnetization curve obtained in the framework of the Weiss molecular-field approximation to the Heisenberg model^16^ (See Supplementary Note). For the localized Gd 4*f* moments, we assume a pure spin moment of J = S = 7/2. Neglecting the kink at 60 K, which we will discuss later on, the curve perfectly fits the ARPES data. This supports our finding from the band structure calculation, that the band splitting is mediated via exchange coupling to the localized Gd 4*f* moments. The fit confirms, that the splitting indeed vanishes at a temperature of ~90 K and saturates at ~160 meV at the considered point of the Brillouin zone. As we have already mentioned, the spin splitting of the Shockley state is highly anisotropic.

In Fig. 5 we also show the temperature-dependent spin splitting for the Dirac cone. Here, the splitting can be well resolved below ~60 K. The overall splitting is less than half as large as for the Shockley state. Therefore, similar to the Shockley state, an evaluation of the splitting remains inaccessible as soon as it drops below ~50 meV already slightly above 60 K, i.e. at even lower temperatures. Furthermore, as the Dirac cone has mainly Rh 4*d* character, spin-orbit coupling (SOC) has to be taken into account, which mixes with the exchange splitting. From our calculations, we estimate the spin-orbit splitting to be of the order of 40–45 meV for the Dirac cone close to the 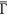-point, which is of the same order as the observed splitting around 60 K. As SOC does not depend on temperature, it might dominate the splitting at temperatures above ~65 K and even remain above T_N_. Ignoring all SOC effects, we nevertheless tried to fit the Dirac cone splitting in the same Weiss theory framework as the Shockley state and interestingly find the same critical temperature of ~90 K as in the previous case. Our findings imply that the spin splitting observed by ARPES is driven by the fundamental magnetic exchange interaction, which in the case of the Dirac cone might be complicated by SOC and its resonant nature, where more than one ordered Gd layer is involved in the interplay.

### Magnetic properties in the bulk and at the surface from XMLD

**Figure 6:**
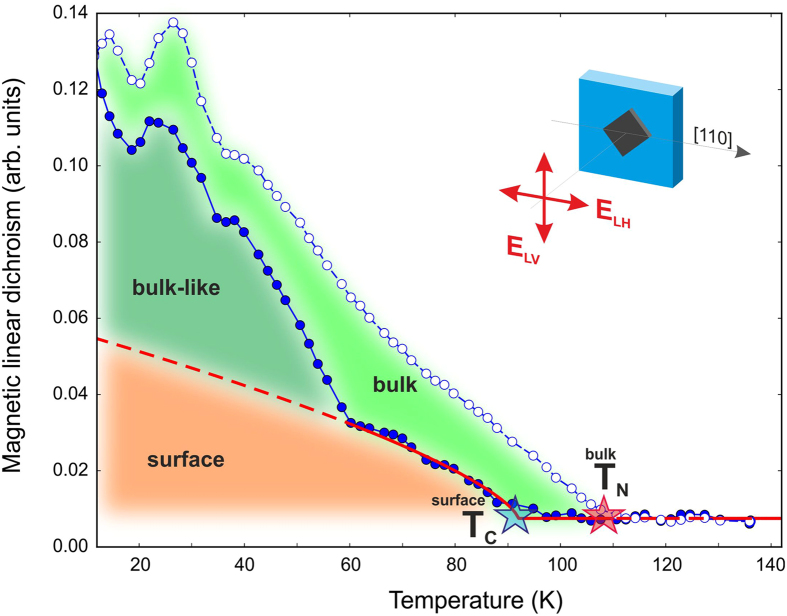
XMLD insight into the ordering of Gd 4*f* moments in the bulk and at the subsurface. Temperature dependence of the XMLD signal in fluorescence (TFY, open symbols) and in total electron yield (TEY, solid symbols). TFY probes the bulk magnetization, whereas TEY is more sensitive to the surface region. The dashed lines are guides to the eyes. The red solid line shows the fit result by means of the Weiss molecular-field approximation to the Heisenberg model^16^. The inset schematically illustrates the experimental geometry for the XMLD experiment.

To shed further light on the temperature-dependent spin splitting of the 2DESs, which is directly linked to the ordering of the Gd 4*f* moments, we performed X-ray magnetic linear dichroism (XMLD) experiments at the Gd M_5_ (3*d* → 4*f*) absorption edge. XMLD is sensitive to both FM and AFM order because the measured signal is proportional to the square of the ordered magnetic moment 〈**M**^**2**^〉^24^,^25^. Using XMLD, we detected the onset of magnetic order in the surface region and in the bulk of GdRh_2_Si_2_ by simultaneously looking at the total electron yield (TEY) and the total fluorescence yield (TFY) signal, respectively^26^. The TEY signal probes less than 20–30 Å near the surface with the main contribution coming from the first Gd layer^27^. In TFY the probing depth is of the order of 100 nm and thus an almost pure bulk signal is seen. In Fig. 6 we plot the results of our XMLD measurements. The magnetic part of the linear dichroism was separated from the natural linear dichroism by subtracting the XLD signal measured at 140 K from all spectra. From the TFY data (open symbols), the onset of AFM order in the bulk can be seen at ~107 K in perfect agreement with the bulk Néel temperature determined from macroscopic measurements^4^. However, from the TEY signal (solid symbols) it becomes evident that the magnetic order at the surface sets in substantially below the bulk Néel temperature - at around 90 K, which agrees well with the onset of the Shockley state splitting seen in ARPES (see Fig. 5). Furthermore, the TEY curve reveals an interesting kink-like feature near 60 K, like in the spin splitting curve derived from our ARPES measurements. From the difference of the TFY and TEY curves above ~60 K we may assume, that in this temperature range the magnetism at the surface is mainly mediated by the Shockley state, and is different from that in the bulk. Below ~60 K, the slope and shape of the TEY curve quickly approache those of the TFY curve. This effect might indicate that in this temperature range a new channel for alignment between 4*f* moments at the surface and those deeper in the bulk has appeared along the *c*-axis. This is perfectly fine with our observation of the spin splitting onset of the Dirac cone, which is a surface resonance state penetrating deeper in the bulk. Thus, the Dirac cone state might open an additional exchange channel leading to an accelerated growth of macroscopic magnetic domains - ordered Gd 4*f* moments near the surface. In that regard we may propose that below ~60 K the magnetic systems at the surface and in the bulk become linked together and further growth of magnetic domains takes place simultaneously upon cooling at the surface and in the bulk. It should be noted that the XMLD signal is an average over aligned moments within the beam spot size S_beam_ and thus also depends on the domain size S_domain_ if S_domain_ < S_beam_ = 100 × 20 μm^2^. We believe that the dynamics of the domain growth and the role of the Dirac cone might be an interesting challenge for further studies on GdRh_2_Si_2_, a material which offers a rich playground for investigating the magnetic interplay between localized and itinerant electrons at the surface and in the bulk. One essential remark needs to be made about the XMLD experiment. In contrast to ARPES measurements, there is no direct evidence that during our XMLD measurements we have exclusively investigated the Si-terminated surface of GdRh_2_Si_2_. Nevertheless, the obtained results are meaningful and important when combined with the ARPES data. First, the T-dependence of the measured XMLD signals in TEY (surface) and TFY (bulk) modes are different, implying rather different magnetic properties near the surface and in the bulk. Second, the onset of the TEY signal is seen at ~90 K which is in fine agreement with the onset of the splitting of the Shockley state of the Si-terminated surface seen in ARPES. Third, the “kink” seen in the T-dependence of the TEY signal appears at the same temperature of about 60 K, where the splitting of the resonant Dirac cone band has been detected. We therefore believe that the XMLD data reflect the magnetism of GdRh_2_Si_2_ in the bulk and, at least to a large part, at the Si-terminated surface. It is possible that within the 100 by 20 microns spot size there are also Gd-terminated parts of the surface. One may assume that those could contribute to the constant background seen in our experiment.

We now compare the essential differences in properties of EuRh_2_Si_2_ and GdRh_2_Si_2_. It is worth noting that in EuRh_2_Si_2_ the onset temperature of the Shockley state spin splitting (T_s_)^23^ also differs from the bulk Néel temperature (T_N_ = 24.5 K), but in this case T_S_ is more than 30% larger than T_N_, while in GdRh_2_Si_2_ T_S_ is ~16% below T_N_. We propose that this difference between the two systems might arise from the competition between an enhanced in-plane exchange within the topmost Eu/Gd layer due to the strongly polarized Shockley states on the one hand, and a reduction of the overall magnetic coupling at the surface due to a reduced coordination number on the other hand. The former effect is likely of similar strength in both systems, since the spin splitting of the Shockley states is similar in both Eu- and Gd-based materials. On the other hand the “reduced coordination” effect is expected to be much stronger in the Gd- than in the Eu-based compound, because the exchange coupling between adjacent rare earth layers deep in the bulk is very strong in GdRh_2_Si_2_, whereas it is rather weak in EuRh_2_Si_2_. This is evidenced by the magnitude of the field required to get saturation magnetization, i.e. to rotate the ferromagnetic rare earth layers from AFM to FM stacking. This field is huge in GdRh_2_Si_2_, about 50 T^4^, but tiny in EuRh_2_Si_2_, merely about 0.1 T applied along the basal plane^28^. Thus EuRh_2_Si_2_ is already a quasi-two-dimensional magnetic system in the bulk, while GdRh_2_Si_2_ is truly three-dimensional. Altogether, at the surface of EuRh_2_Si_2_ the ordering temperature is only slightly pushed down by the removal of adjacent Eu layers, but strongly pushed up by the additional exchange, mediated through the Shockley states, thus T_S_ > T_N_. In contrast, in GdRh_2_Si_2_ the removal of adjacent Gd layers pushes the ordering temperature significantly down, while this effect cannot be compensated by the additional in-plane exchange, thus T_S_ < T_N_. The much stronger coupling along the *c*-direction in the Gd-based compound is likely due to the hybridization provided by the Gd 5*d* electrons.

**Figure 7:**
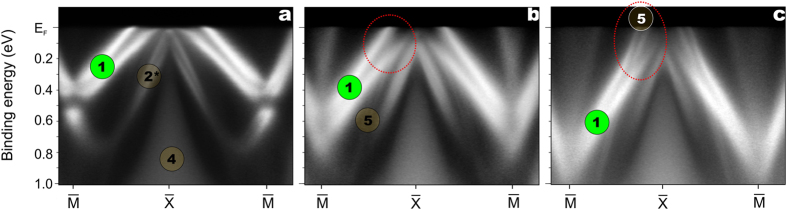
Modification of the itinerant magnetism at the Si-terminated surface by K-deposition. ARPES data taken from a Si-terminated GdRh_2_Si_2_ sample using 55 eV photons at 50 K for a freshly cleaved surface (**a**) and after deposition of ~0.5 ML (**b**) and ~1.3 ML (**c**) of potassium on its top at 50 K. The measurements were performed along the 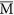-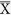-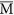 direction. The surface and bulk related spectral features are labelled in accordance to the theoretically derived bands seen in Figs 2 and 3. The dotted ellipse highlights the spin-dependent hybridization of the 2D band (1) with the bands (5).

Our combined experimental and theoretical studies of GdRh_2_Si_2_ show that its silicon-terminated surface reveals rich and unique magnetic properties below ~90 K. It bears two distinct 2DESs arising from Shockley (surface state) and Dirac fermions (surface resonance state). Both are subject to strong exchange interaction with the hidden and magnetically active 4*f* moments of Gd. This interaction lifts up the spin degeneracy of both electron states leading to the appearance of spin-split subbands with largest splitting values of 185 meV and 70 meV for the Shockley and Dirac state, respectively. Exploring the temperature evolution of the ordering of the 4*f* moments at the surface we find that between ~90–60 K surface and bulk magnetism behave independently. Below ~60 K, the resonant Dirac cone state seems to link the surface and bulk magnetic subsystems. Evidently, this material still leaves plenty of unanswered questions and offers a rich playground for studying magnetic phenomena at silicon-terminated surfaces coupled to magnetically active overlayers. The surface magnetism of this compound might be further linked to a functional surface layer of organic molecules or ordered magnetic materials with metallic or semiconducting properties. To demonstrate this, we deposited potassium onto a freshly cleaved Si-terminated surface of GdRh_2_Si_2_. The respective data are shown in Fig. 7. As a result a systematic shift of the spin-split Shockley surface state to higher binding energies is observed that is caused by charge transfer from K into this state. Its spin splitting not only survives upon K deposition, but one can clearly see that the spin-split bands labelled as (1) strongly interfere and hybridize with the bands marked as (5) lying at the periphery of the 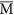-gap. Additionally, a new parabolic band appears at the 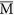
**-**point and touches the Shockley state that is possibly derived from a respective unoccupied surface state of the clean Si-terminated surface (see Fig. 2). The observed surface electron doping effects together with the spin-derived hybridization lead to essential modifications of the 2DESs implying also changes of the magnetic properties at the topmost layers of the material. In the end we would like to add that previously, we have demonstrated that the Dirac fermion states can couple with ultra-heavy quasiparticles in crystalline 4*f*-based systems^8^, i.e. couple with the electronic degree of freedom of 4*f* electrons, while here, we show that the Dirac fermions may also couple via exchange to the magnetic degree of freedom of 4*f* electrons.

## Methods

### Experiment

ARPES studies were carried out at the Swiss Light Source (SIS X09LA instrument), the Diamond Light Source (I05 beamline) and BESSY-II (One-cubed ARPES instrument) and are described in details elsewhere^8^,^23^. The ARPES spectra were acquired using a Scienta R4000 electron energy analyzer. The overall energy and angular resolutions were 10 meV and 0.1 degree, respectively. High quality single-crystalline samples of GdRh_2_Si_2_^4^ were cleaved *in situ* in ultra-high vacuum at a base pressure better than 8 × 10^−11 ^mbar. Surface regions terminated by a Si layer were selected as the beam was scanning across the sample by looking at the ARPES map in the vicinity of the 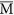
**-**point of the surface Brillouin zone, where the Shockley state settled in a gap of bulk-projected bands as a characteristic of Si termination was observed. The surface origin and two-dimensional character of the electron state at the 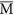-point were additionally confirmed by h*ν*-dependent measurements, which indicate the absence of any dispersion along the **k**_**z**_- direction. The beam spot size was set to 20 × 80 μm^2^. The temperature-dependent measurements were always performed going from high to low temperatures in order to avoid fast sample aging.

XMLD experiments at the Gd M_5_ edge were carried out at the European Synchrotron Radiation Facility (soft X-ray beamline ID32) using the high-field magnet end station^29^. The samples were cleaved in UHV at 140 K in the high-field magnet immediately prior to the measurements. A small field of 100 mT was applied along the (110) axis of the crystal which was aligned to the **E** vector of the incident light for linear horizontal polarization. The temperature dependence of the XMLD was measured by slowly ramping the temperature down to 10 K and back up to 140 K and continuously measuring the absorption with linear horizontal (LH) and linear vertical (LV) polarization, simultaneously detecting the total electron yield and total fluorescence yield from the sample. The beam spot size at the sample was set to 100 × 20 μm^2^.

### Theory

*Ab initio* calculations were performed within the projector augmented-wave method^30^ (VASP code^31^,^32^) using the generalized gradient approximation (GGA) to the exchange-correlation potential^33^. The Hamiltonian contained scalar-relativistic corrections and spin-orbit coupling was taken into account by a second variation procedure^34^. We set the energy cutoff for the plane-wave expansion of wave functions to 256.5 eV and sampled the two-dimensional Brillouin zone with a 12 × 12 × 1 **k**-point grid. In order to correctly describe the Gd 4*f* and Rh 4*d* states, we used the GGA + U approach^35^,^36^. For the Gd 4*f* electrons values of U = 6.7 eV and J = 0.7 eV were chosen, while the Rh 4*d* electrons were treated with U = 3.5 eV and J = 0.6 eV. The GdRh_2_Si_2_ (001) surface was simulated by a 32-layer-thick asymmetric slab with the topmost (lowermost) surface terminated by Gd (Si).

## Additional Information

**How to cite this article**: Güttler, M. *et al*. Robust and tunable itinerant ferromagnetism at the silicon surface of the antiferromagnet GdRh_2_Si_2_. *Sci. Rep*. **6**, 24254; doi: 10.1038/srep24254 (2016).

## Supplementary Material

Supplementary Information

## Figures and Tables

**Figure 1:**
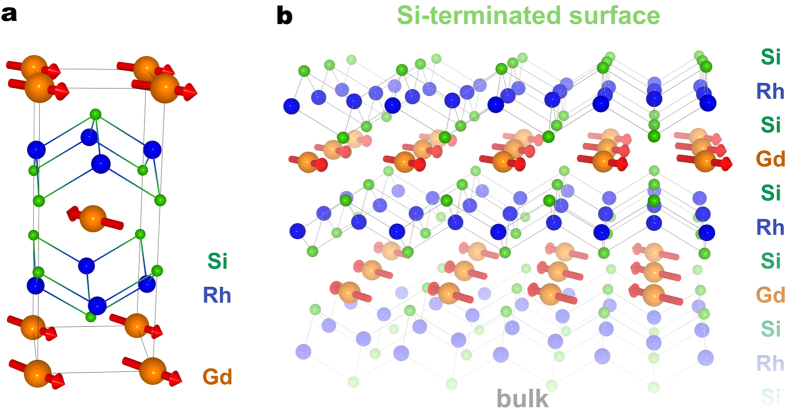
Schematical structure of GdRh_2_Si_2_ with AFM ordering. (**a**) Tetragonal crystal structure with red arrows indicating the Gd 4*f* moments and (**b**) Si-terminated surface of GdRh_2_Si_2_; the Si-Rh-Si blocks separate antiferromagnetically stacked Gd layers. The in-plane ordering of the Gd 4*f* moments is ferromagnetic^4^,^6^.

## References

[b1] Jansen R. . Silicon spintronics with ferromagnetic tunnel devices. Semicond. Sci. Technol. 27, 083001 (2012).

[b2] Getzlaff M. . Temperature-dependent exchange splitting of the magnetic Gd(0001) surface state. J. Mag. Mag. Mat. 184, 155 (1998).

[b3] Patil S. . Crystalline electric field splitting of 4*f* states in YbIr_2_Si_2_: an ARPES view. JPS Conf. Proc. 3, 011001 (2014).

[b4] Kliemt K. & Krellner C. Single crystal growth and characterization of GdRh_2_Si_2_. J. Crys. Growth 419, 37 (2015).

[b5] Felner I. & Nowik I. Local and itinerant magnetism and superconductivity in RRh_2_Si_2_ (R = rare earth). Solid State Commun. 47, 831 (1983).

[b6] Slaski M., Leciejewicz J. & Szytula A. Magnetic ordering in HoRu_2_Si_2_, HoRh_2_Si_2_, TbRh_2_Si_2_ and TbIr_2_Si_2_ by neutron diffraction. J. Mag. Mag. Mat. 39, 268 (1983).

[b7] Vyalikh D. V. . Tuning the dispersion of 4*f* -bands in the heavy-fermion material YbRh_2_Si_2_. J. of El. Spec. Rel. Phen. 181, 70 (2010).

[b8] Höppner M. . Interplay of Dirac fermions and heavy quasiparticles in solids. Nature Commun. 4, 1646 (2013). 23552061 10.1038/ncomms2654

[b9] Hüfner S. Photoelectron Spectroscopy. Principles and Applications. Third Edition. (Springer-Verlag: Berlin Heidelberg New York, , 1995).

[b10] Tamm I. E. A possible kind of electron binding on crystal surfaces. Z. Phys. 76, 849 (1932).

[b11] Shockley W. On the Surface States Associated with a Periodic Potential. Phys. Rev. 56, 317 (1939).

[b12] Gartland P. O. & Slagsvold B. J. Transitions conserving parallel momentum in photoemission from the (111) face of copper. Phys. Rev. B 12, 4047 (1975).

[b13] Heimann P., Neddermeyer H. & Roloff H. F. Ultraviolet photoemission from intrinsic surface states of the noble metals. J. Phys. Condens. Matter 10, L17 (1977).

[b14] Reinert F. & Hüfner S. Photoemission spectroscopy—from early days to recent applications. New J. Phys. 7, 97 (2005).

[b15] Davison S. G. & Steslicka M. Basic Theory of Surface States. Monographs on the physics and chemistry of materials. (Clarendon Press. Oxford, 1992).

[b16] Getzlaff M. Fundamentals of magnetism. (Springer-Verlag: Berlin Heidelberg New York, , 2008).

[b17] Molotkov S. N. On the specificity of the electron spectrum of two-dimensional lattices. JETP Lett. 90, 339–345 (2009).

[b18] Molotkov S. N. On the electronic spectrum of low-dimensional structures with the symmetry of borders. JETP Lett. 94, 282–287 (2011).

[b19] Vyalikh D. V. . **k**-dependence of the crystal-field splittings of 4*f* states in rare-earth systems. Phys. Rev. Lett. 105, 237601 (2010). 21231502 10.1103/PhysRevLett.105.237601

[b20] Vyalikh D. V. . Tuning the Hybridization at the Surface of a Heavy-Fermion System. Phys. Rev. Lett. 103, 137601 (2009). 19905540 10.1103/PhysRevLett.103.137601

[b21] Danzenbächer S. . Insight into the f-Derived Fermi Surface of the Heavy-Fermion Compound YbRh_2_Si_2_. Phys. Rev. Lett. 107, 267601 (2011). 22243181 10.1103/PhysRevLett.107.267601

[b22] Güttler M. . Tracing the localization of 4*f* electrons: ARPES on YbCo_2_Si_2_, the stable trivalent counterpart of the heavy-fermion YbRh_2_Si_2_. Phys. Rev. B 90, 195138 (2014).

[b23] Chikina A. . Strong ferromagnetism at the surface of an antiferromagnet caused by buried magnetic moments. Nat. Commun. 5, 3171 (2014). 24445395 10.1038/ncomms4171

[b24] Thole B. T. . Strong Magnetic Dichroism Predicted in the *M*_4,5_ X-Ray Absorption Spectra of Magnetic Rare-Earth Materials. Phys. Rev. Lett. 55, 2086–2088 (1985). 10032006 10.1103/PhysRevLett.55.2086

[b25] Alders D. . Temperature and thickness dependence of magnetic moments in NiO epitaxial films. Phys. Rev. B 57, 11623–11631 (1998).

[b26] Stöhr J. NEXAFS Spectroscopy. Springer Series in Surface Science. (Springer-Verlag: Berlin Heidelberg New York, , 1992).

[b27] Frazer B. H. . The probing depth of total electron yield in the sub-keV range: TEY-XAS and X-PEEM. Surf. Sci. 537, 161–167 (2003).

[b28] Seiro S. & Geibel C. From stable divalent to valence-fluctuating behavior in Eu(Rh_1−x_Ir_x_)_2_Si_2_ single crystals. J. Phys. Condens. Matter. 23, 375601 (2011). 21878716 10.1088/0953-8984/23/37/375601

[b29] Kummer K. . The high-field magnet endstation for X-ray magnetic dichroism experiments at ESRF soft X-ray beamline-ID32. J. Synchrotron Rad. 23, 464 (2016). 10.1107/S160057751600179X PMC5297906 26917134

[b30] Blöchl P. E. Projector augmented-wave method. Phys. Rev. B 50, 17953–17979 (1994). 10.1103/physrevb.50.17953 9976227

[b31] Kresse G. & Furthmüller J. Efficient iterative schemes for *ab initio* total-energy calculations using a plane-wave basis set. Phys. Rev. B 54, 11169–11186 (1996). 10.1103/physrevb.54.11169 9984901

[b32] Kresse G. & Joubert D. From ultrasoft pseudopotentials to the projector augmented-wave method. Phys. Rev. B 59, 1758–1775 (1999).

[b33] Perdew J. P. . Generalized Gradient Approximation Made Simple. Phys. Rev. Lett. 77, 3865–3868 (1996). 10062328 10.1103/PhysRevLett.77.3865

[b34] Koelling D. D. & Harmon B. N. A technique for relativistic spin-polarised calculations. J. Phys. C: Sol. St. Phys. 10, 3107–3114 (1977).

[b35] Anisimov V. I. . Band theory and Mott insulators: Hubbard U instead of Stoner I. Phys. Rev. B 44, 943–954 (1991). 10.1103/physrevb.44.943 9999600

[b36] Dudarev S. L. . Electron-energy-loss spectra and the structural stability of nickel oxide: An LSDA+U study. Phys. Rev. B 57, 1505–1509 (1998).

