# Supplementary material for: Robust and tunable itinerant ferromagnetism at the silicon surface of the antiferromagnet GdRh2Si2
Source: Sci Rep. 2016 Apr 7;6:24254. doi: 10.1038/srep24254 (PMC4823662; doi:10.1038/srep24254)
Supplement: Supplementary Information [file srep24254-s1.pdf]

Supplementary Information

Robust and tunable itinerant ferromagnetism at the silicon surface of the antiferromagnet $GdRh_2Si_2$

M. Güttler[1,2], A. Generalov[3], M. M. Otrokov[4,5], K. Kummer[6], K. Kliemt[7], A. Fedorov[8], A. Chikina[1], S. Danzenbächer[1], S. Schulz[1], E. V. Chulkov[4,5,12], Yu. M. Koroteev[5,13], N. Caroca-Canales[9], M. Shi[10], M. Radovic[10,11], C. Geibel[9], C. Laubschat[1], P. Dudin[14], T. K. Kim[14], M. Hoesch[14], C. Krellner[7] and D. V. Vyalikh[1,4,12,15]

[1] Institute of Solid State Physics, Dresden University of Technology, Zellescher Weg 16, D-01062 Dresden, Germany

[2] CSNSM, University Paris-Sud and CNRS/IN2P3, Btiments 104 et 108, 91405 Orsay, France

[3]MAX-Laboratory, Lund University, Box 118, 22100 Lund, Sweden

[4] Donostia International Physics Center (DIPC),
Departamento de Fisica de Materiales and CFM-MPC UPV/EHU, 20080 San Sebastian, Spain

[5]Tomsk State University, Lenina Av., 36, 634050 Tomsk, Russia

[6] European Synchrotron Radiation Facility, 71 Avenue des Martyrs,
Grenoble, France

[7] Kristall- und Materiallabor, Physikalisches Institut, Goethe-Universität Frankfurt, Max-von-Laue Straße 1, 60438 Frankfurt am Main, Germany

[8] IFW Dresden, P.O. Box 270116, Dresden D-01171, Germany

[9] Max Planck Institute for Chemical Physics of Solids, Nöthnitzer Strasse 40, 01187 Dresden, Germany

[10] Swiss Light Source, Paul Scherrer Institute, CH-5232 Villigen-PSI, Switzerland

[11]SwissFEL, Paul Scherrer Institut, CH-5232 Villigen PSI, Switzerland

[12]Saint Petersburg State University, Saint Petersburg 198504, Russia

[13]Institute of Strength Physics and Materials Science, RAS, 634021 Tomsk, Russia

[14] Diamond Light Source, Didcot OX11 0DE, UK.

[15] IKERBASQUE, Basque Foundation for Science, 48011 Bilbao, Spain

krellner@physik.uni-frankfurt.de
denis.vyalikh@tu-dresden.de

Supplementary Note:

The magnetization curve used to fit the temperature-dependent spin splitting has been deduced from the conventional Weiss mean-field treatment of the ferromagnetic Heisenberg model [16]. Within the Weiss theory, the magnetization M(T) as a function of temperature is given by

$$\frac{M}{M_S} = B_J\left(\frac{3S}{S+1}\frac{M}{M_S}\frac{T_C}{T}\right),$$

where B_J is the Brillouin function. The saturation magnetization M_S and critical temperature T_C were used as fitting parameters. The total spin S has been set to 7/2. From our fit, we obtained an anisotropic saturation splitting of 157 meV for the Shockley state and 67 meV for the Dirac state, respectively, for the used Brillouin zone cuts.

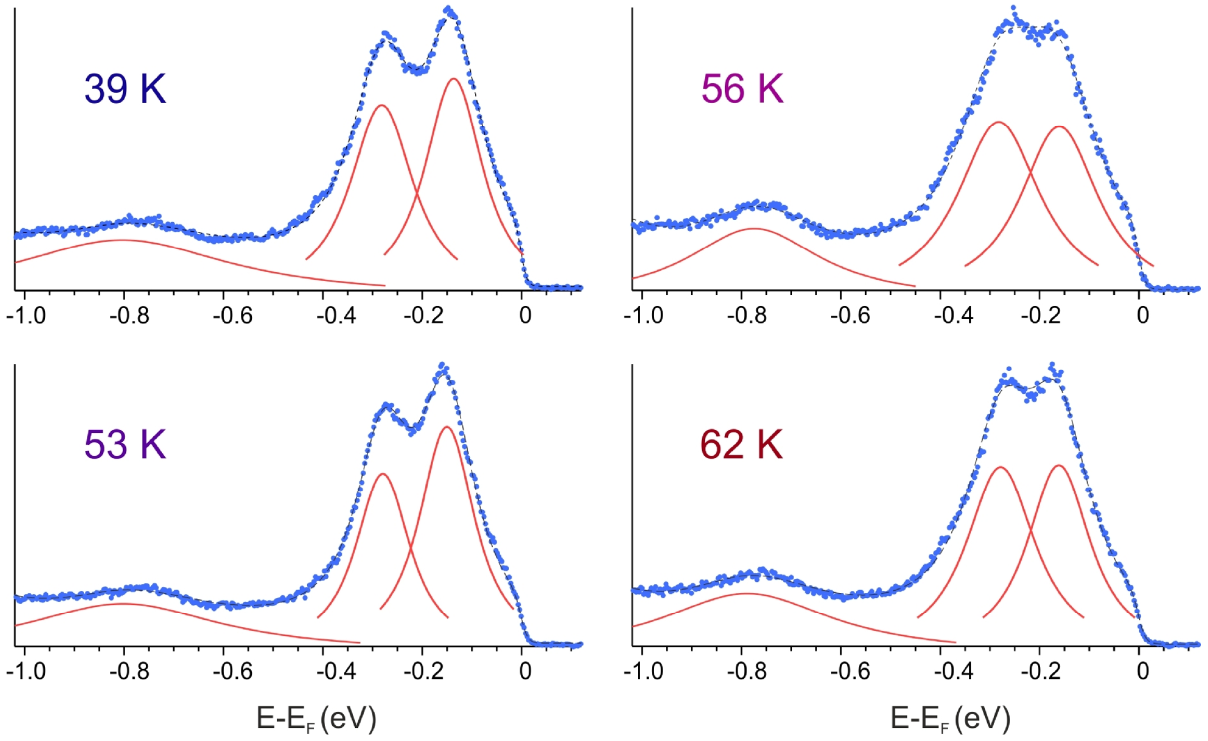

Supplementary Figure S1: Peak fits of the EDC indicated as white line in Fig. 3b for four exemplary temperatures. The EDCs (blue dots) were fitted with a sum of three Voigt line shapes (red lines), two for the exchange-split $\overline{M}$ surface state and one for the broad peak at higher binding energy. After adding a weak quadratic background, we multiplied the fit function (dashed black line) with a Fermi-Dirac distribution broadened by the experimental temperature and the instrumental resolution. The exchange splitting of the Shockley state around the $\overline{M}$-point is well resolved at lower temperatures (39 and 53 K). At temperatures slightly below 60 K, where the kink appears in the T dependence of the splitting (Fig. 5), the peaks appear to broaden and the splitting gets blurred, whereas it reappears at higher temperatures (62 K).
